# Exoproteome and Secretome Derived Broad Spectrum Novel Drug and Vaccine Candidates in *Vibrio cholerae* Targeted by *Piper betel* Derived Compounds

**DOI:** 10.1371/journal.pone.0052773

**Published:** 2013-01-30

**Authors:** Debmalya Barh, Neha Barve, Krishnakant Gupta, Sudha Chandra, Neha Jain, Sandeep Tiwari, Nidia Leon-Sicairos, Adrian Canizalez-Roman, Anderson Rodrigues dos Santos, Syed Shah Hassan, Síntia Almeida, Rommel Thiago Jucá Ramos, Vinicius Augusto Carvalho de Abreu, Adriana Ribeiro Carneiro, Siomar de Castro Soares, Thiago Luiz de Paula Castro, Anderson Miyoshi, Artur Silva, Anil Kumar, Amarendra Narayan Misra, Kenneth Blum, Eric R. Braverman, Vasco Azevedo

**Affiliations:** 1 Centre for Genomics and Applied Gene Technology, Institute of Integrative Omics and Applied Biotechnology (IIOAB), Nonakuri, Purba Medinipur, West Bengal, India; 2 Department of Biosciences and Biotechnology, School of Biotechnology, Fakir Mohan University, Jnan Bigyan Vihar, Balasore, Orissa, India; 3 School of Biotechnology, Devi Ahilya University, Indore, India; 4 Unit for research, School of Medicine, Autonomous University of Sinaloa, Cedros y Sauces, Fracc. Fresnos, Culiacan, Mexico; 5 Laboratório de Genética Celular e Molecular, Departamento de Biologia Geral, Instituto de Ciências Biológicas (ICB), Universidade Federal de Minas Gerais, Pampulha, Belo Horizonte, Minas Gerais, Brazil; 6 Instituto de Ciências Biológicas, Universidade Federal do Pará, Belém, Para, Brazil; 7 Center for Life Sciences, School of Natural Sciences, Central University of Jharkhand, Ranchi, Jharkhand State, India; 8 College of Medicine, University of Florida, Gainesville, Florida, United States of America; 9 Center for Clinical and Translational Science, College of Medicine, University of Vermont, Burlington, Vermont, United States of America; 10 Weill-Cornell College of Medicine, Cornell University, New York, New York, United States of America; University of Delhi, India

## Abstract

*Vibrio cholerae* is the causal organism of the cholera epidemic, which is mostly prevalent in developing and underdeveloped countries. However, incidences of cholera in developed countries are also alarming. Because of the emergence of new drug-resistant strains, even though several generic drugs and vaccines have been developed over time, *Vibrio* infections remain a global health problem that appeals for the development of novel drugs and vaccines against the pathogen. Here, applying comparative proteomic and reverse vaccinology approaches to the exoproteome and secretome of the pathogen, we have identified three candidate targets (*ompU*, *uppP* and *yajC*) for most of the pathogenic *Vibrio* strains. Two targets (*uppP* and *yajC*) are novel to *Vibrio*, and two targets (*uppP* and *ompU*) can be used to develop both drugs and vaccines (dual targets) against broad spectrum *Vibrio* serotypes. Using our novel computational approach, we have identified three peptide vaccine candidates that have high potential to induce both B- and T-cell-mediated immune responses from our identified two dual targets. These two targets were modeled and subjected to virtual screening against natural compounds derived from *Piper betel*. Seven compounds were identified first time from *Piper betel* to be highly effective to render the function of these targets to identify them as emerging potential drugs against *Vibrio*. Our preliminary validation suggests that these identified peptide vaccines and *betel* compounds are highly effective against *Vibrio cholerae*. Currently we are exhaustively validating these targets, candidate peptide vaccines, and *betel* derived lead compounds against a number of *Vibrio* species.

## Introduction

*Vibrio cholerae* is a noninvasive gram-negative bacterium that causes water borne disease cholera, which is characterized by profuse watery diarrhea and vomiting [1]. The severity of the diarrhea and vomiting causes rapid dehydration and electrolyte imbalance that leads to death. The *V. cholerae O395* strain is a classical O1 serotype strain responsible for cholera epidemics in Asian countries, and the non-O1 sero-group *Vibrio cholerae O139* has been implicated as the causative agent of sporadic cases of gastro-enteritis and extra-intestinal infections [2], [3]. Both of the strains have been reported to cause significant numbers of morbidities [4], [5]. Although considerable research is ongoing to develop new drugs and vaccines and many antibiotics are already used to treat cholera, the infection remains frequently uncontrolled because of emerging antibiotic resistance of the pathogen [6], [7], [8]. Therefore, novel drugs and vaccines must be developed to tackle the *Vibrio* infection and transmission.

The identification of antigenic and virulence factors is paramount in developing antibiotics against a pathogen. In most cases, exomembrane (surface exposed) and secretary proteins exhibit antigenicity and virulence and are therefore suitable for targeting. Similarly, in the post-genomics era, computational approaches for the identification of genomic targets [9] and the use of reverse vaccinology [10] are becoming popular for rapid identification of novel targets to develop both drugs and vaccines against any given pathogen.

The present study aims to identify broad spectrum and novel drug and vaccine targets for a number of *Vibrio* strains, including *V. cholerae* strains O395 and *O139*; to design peptide vaccines; and to identify lead natural compounds first time from *Piper betel*, a well-known plant with medicinal value, to make use of these targets.

## Materials and Methods

### Drug and Vaccine Target Prioritization Parameters

Target prioritization in pathogenic microorganisms is accomplished in various ways [11]. Among these important prioritization considerations are subcellular localization, non-host homolog essential genes, core pathogen genes, pathogenic island association, involvement in the pathogen’s unique metabolic pathways, druggability, availability of 3D structural information, and low molecular weight of the target protein (≤110 kDa) [9], [11]–[14]. The exoproteome and secretome are good source of targets for developing vaccines and drugs (dual targets) [9], [10]. Therefore, we first screened the exoproteome and secretome of the pathogen for potential targets, followed by the application of other prioritization parameters to identify targets.

### Screening of the Exoproteome and Secretome and Target Identification

We applied the classical reverse vaccinology strategy [10] and a modified method of subtractive proteomics [15], [16] to identify candidate drug vaccine targets in *V. cholerae* strain *O395* and other *Vibrio* serotypes. In brief, the *Vibrio cholera O395* proteome, which consists of 3875 proteins, was screened using CELLO [17], PSLpred [18], PSORTb [19], SOSUI-GramN [20], and SurfG^+^ [21] to identify the exomembrane and secreted proteins. Thereafter, the essential, non-human homolog *Vibrio* proteins (putative targets) from the pool of exoproteome and secretome were identified using the Database of Essential Genes (DEG) [22] and NCBI BLASTp [23], as described by Barh *et al.,* 2011 [15]. Selected non-human homolog essential *Vibrio* proteins were then checked for their pathway involvement, and the best targets were selected based on the involvements of these targets in the unique essential bacterial metabolic pathways and another twelve criteria as described by Barh *et al.,* 2011 [15] for target selection. The final list of identified targets was then checked for their presence in different *Vibrio* strains and related species using NCBI prokaryotic genome BLASTp.

### Additional Evaluation of the Essentiality Parameters of Targets

The DEG-based essentiality of the identified targets was further validated using sequence-based computational approaches: (i) strand-bias; (ii) codon adaptation index (CAI); (iii) patterns of enzyme classes distributed, and (iv) clusters of orthologous groups (COG) of proteins. Essential genes are mostly located on leading strands and show strand bias [24]. We used Ori-Finder [25] to check the replication origin- and replication termini-based determination of strand-bias and the localization of the identified target genes in leading or lagging strands. CAI values are reported as one of the measures to evaluate essential genes, with a CAI >0.5 indicative of significant essentiality [26]. We used ACUA software [26] to calculate the CAI values of our identified targets. The distribution of enzyme classes of the targets was determined with BRENDA [27] and UniProtKB [28]. The targets were also examined for their bias toward COG functional subcategories for essentiality as per the findings of Lin *et al.,* 2010 [29].

### Evaluation of Prioritization Parameters of Targets

We further checked the identified targets for their molecular weight if they are of ≤110 kDa using UniProtKB [28]. The druggability of the targets was determined using the DrugBank database [30]. The amino acid sequences of identified potential targets were aligned using BLASTp with a cutoff *E-value* = 0.01 against the DrugBank -listed targets for which specific compounds are available in the database. The availability of 3D structural information of targets was verified with PDB [31]. When structures were not available, a homology modeling or threading approach was performed and verified with various 3D modeling parameters (see 3D modeling of targets section).

### Additional Prioritization Parameters of Targets by PPIs and Hostpathogen Interactions

Target identification based on host-pathogen interactions has been implemented in many organisms, including *M. tuberculosis* [32]. Therefore, to verify the reliability of our identified targets, we searched for protein-protein interactions (PPIs) among the identified targets and also host-pathogen interactions. All *V. cholera O395* targets were selected to make PPI networks using VisAnt [33]. Further, KEGG pathways [34] were incorporated into the PPI networks and analyzed for their involvement in bacterial pathogenesis and essential pathways. To identify host-pathogen interactions, 20,000 experimentally-validated host-pathogen interactions for 24 pathogens were downloaded from the PathoSystems Resource Integration Center (PATRIC) database [35]. In PATRIC, *Vibrio-*specific host-pathogen interaction data are not available. Therefore, we used sequences from pathogens listed in PATRIC that are 90% homologous to our identified *Vibrio* targets to determine interactions and interacting human counterparts. The interacting human counterparts were also analyzed for their involvement in key biological processes and pathways involved in host response to infection, such as immunity and apoptosis, and examined whether they are key nodes in those pathways using the Search Tool for the Retrieval of Interacting Genes (STRING) [36] and the Database for Annotation, Visualization and Integrated Discovery (DAVID) [37]. Targets that are involved in bacterial pathogenesis or essential pathways and interact with key molecules in host response pathways are generally more effective targets.

### Prediction of Antigenic B-cell Derived T-cell Epitopes

Once the targets are finalized, the novel strategy of epitope designing as described by Barh *et al.,* 2011 [16] was applied to design peptide vaccines from the vaccine targets. Briefly, the secreted and exomembrane proteins were checked for antigenicity using the VaxiJen v2.0 server (threshold = 0.4, ACC output) [38], and thereafter, their virulences were predicted using VirulentPred [39]. Proteins that were antigenic according to VirulentPred and showed an antigenicity score >0.5 in VaxiJen were selected. The exomembrane sequences of each virulent protein commonly derived from VaxiJen and VirulentPred analysis were determined by TMHMM v2.0 [40]. The BCPreds server [41] was used for B-cell epitope prediction (cutoff >0.8, 20-mer epitopes) and epitope sequences were matched with surface-exposed sequences of corresponding proteins. The surface-exposed B-cell epitope sequences were further checked for antigenicity using VaxiJen, and the best epitopes were selected for T-cell epitope prediction using ProPred [42] and ProPred I [43]. QSAR-based simulation analysis of each T-cell epitope was performed by MHCPred v.2 [44] and VaxiJen to detect half maximal (50%) inhibitory concentration (IC_50_) and antigenicity, respectively. For a second level confirmation, the selected T-cell epitopes were further screened by T-epitope designer [45], and epitopes were selected that showed binding affinity to ≥80% of HLA molecules, including the A*0201, A*0204, and B*2705, DRB1*0101 and DRB1*0401 alleles that are most common in the human population. Finally, epitopes that bound more than 13 MHC molecules in ProPred and ProPred-I with less than 100 nM IC_50_ for DRB1*0101 in MHCPred v2.0 and that bound ≥80% of HLA molecules in T-epitope designer were selected for fold-level topology analysis to select the best epitopes.

### 3D Modeling of Targets and Topology Analysis of Epitopes

For topology analysis of the identified epitopes and for virtual screening, the target proteins were modeled. The Phyre2 server [46] was used for homology modeling, and the threading approach was performed using the I-TASSER server [47]. The homology-based models were validated using the Structure Analysis and Verification Server (SAVS) Vs.4 (http://services.mbi.ucla.edu/SAVES/), and threading-based models were based on confidence scores (C-score range −5.0 to +2.0) and TM-scores of the resultant protein models. Further loop refinement of threading-based models was done by the ModLoop server [48], and finally, structure verification was performed by ERRAT plot version 2.0 [49], RAMPAGE [50], and the Dali server [51]. The localization and positioning of the epitopes within the folded proteins were analyzed using Pepitope server [52].

### Ligand Library Preparation and Virtual Screening

*Piper betel*, one of the economic crops of West Bengal, India, is reported to have various medicinal and antimicrobial properties. However, no specific compound from this plant has so far been tested for antibacterial property. We collected 128 natural compounds of *Piper betel* from published literature to construct our ligand library. The library was also enriched with 35 well known antibiotics that are used to treat cholera with an aim to compare the efficacy of *betel* compounds with these antibiotics. The catalytic pockets of identified targets were determined using Molegro Virtual Docker (MVD) [53], CASTp [54], Pocket-Finder [55], and Active Site Prediction Server [56]. GOLD 4.1.2 software [57] was used for virtual screening. The best five *betel* derived ligands and antibiotics based on GOLD fitness scores and negative binding energy were selected and further validated using RMSD and MolDock scores in Molegro Virtual Docker 4.2.0 screening. The efficacy of top five *betel* compounds in respect to the top five antibiotics were determined based on GOLD fitness and Molegro Virtual Docker scores.

### Preliminary Validation of Epitopes using IEDB and Betel Compounds against *V. cholerae O1 Inaba*

We preliminary validated the identified candidate peptide vaccines using the Immune Epitope Database (IEDB) [58]. One of the identified candidate *betel* compounds was also checked for its anti-*Vibrio* properties against *V. cholerae O1 Inaba*. The bacteria were maintained in Mueller-Hinton (MH) Broth, placed on a shaker incubator and grown at 37°C for 16–18 h, to reach the logarithmic phase. After that, bacterial cultures were adjusted to an absorbance of 0.1 at 600 nm (1×10^7^ UFC/ml) to test the bactericidal activity of the candidate *betel* compound by two methods. **a)** *Disk diffusion method*: MH agar plates were prepared and spreaded with 1×10^7^ UFC of bacterial cultures, and then sensi-disks (Ampicillin and Chloramphenicol) and disks impregnated with the *betel* compound (dissolved in water, at concentrations of 20, 40, 60, 80, 100, 200 and 300 mM); were placed on MH agar plates and were incubated at 37°C for 24 h. Finally, the zone inhibition was measured by using a Vernier caliper. To test the comparative efficacy of the candidate *betel* compound in respect to conventional anti-Vibrio antibiotics, we performed **b)** *Colony-forming units (CFU/ml) assay*: Here, 1×10^7^ UFC/ml of bacterial suspension were resuspended in tubes containing MH broth, alone (control for bacterial growth) or incubated with 100 µg/ml of Chloramphenicol (control for bacterial inhibition), or with 20, 40, 60, 80, and 100 mM of candidate *betel* compound. After that, tubes were incubated at 37°C for 0, 20, 40, 60 and 80 min in shaking. Finally, the number of viable bacteria was counted each time by obtaining the CFU/ml from serial 10-fold dilutions prepared in MH broth and plating onto MH agar.

## Results and Discussion

### Genome Screening and Target Identification

We identified 513 membrane (160 from Ch-I and 353 from Ch-II) and 317 secreted (113 from Ch-I and 204 from Ch-II) proteins for a total of 830 proteins based on our exoproteome and secretome analysis of *V. cholerae* strain *O395*. The *V. cholerae* strain *O395* proteome consists of 3875 proteins; therefore, 13.2% and 8.18% of proteins of the entire *Vibrio* proteome constitutes exoproteome and secretome, respectively. DEG-based essential gene analysis revealed only 178 essential proteins (119 exomembrane and 59 secreted) out of the total 830. Only 10 essential proteins (7 exomembrane and 3 secreted) were found to be non-human homologs and therefore probable targets **(Table S1)**.

As shown in **Table S2**, among these 10 proteins, 3 are hypothetical (*VC0395_0360*, *VC0395_A1375*, and *VC0395_A2856*). The antigenicity and virulence analysis showed that 9 out of these 10 proteins are antigenic and that 7 are virulent. All 10 proteins were further analyzed for their involvement in the pathogen’s essential unique pathways using the KEGG pathway database. The three hypothetical proteins and *LysE* did not show any pathway involvement and therefore were removed from the analysis. *fadL-3* (Long chain fatty acid transport protein) does not show any vital role in any bacterial essential pathway and was therefore also eliminated. *rodA* (rod shape determining protein) is involved in the regulation of cell shape processes [59] and is essential for *Vibrio*; however, it is not an essential gene for *S. aureus* [60] and also did not provide any T-cell epitopes in further analysis.

Cell membrane-localized *TatC* (sec-independent translocase protein) was identified as an interesting target in *Vibrio*. *TatC* is a virulent protein and is involved in pathways such as membrane transport and the bacterial secretion system. *TatC* has been reported as a target in *M. leprae* [61] and *Klebsiella pneumonia MGH78578* [62]. However, according to the AEROPATH Target Database (http://aeropath.lifesci.dundee.ac.uk/), in *P. aeruginosa*, *TatC* is not an essential gene and it also did not generate any B-cell derived T-cell epitopes in further analysis.

The secreted protein *ompU*/*VC0395_A0162* (Outer membrane protein *ompU*) was found to be an important target as it is involved in the *V. Cholerae* pathogenic cycle. *ompU* is involved in host cell invasion during *Vibrio* infections [63], and for pathogenic *Vibrio harveyi SF-1,* it is reported as a candidate subunit and DNA vaccine [64].

The second most important target is membrane-localized *yajC*/*VC0395_A0472* (Preprotein translocase subunit *yajC*), which is involved in the bacterial secretion system, a vital pathway for bacterial survival. The *C. botulinum yajC* is reported as a putative target [65] and is also listed as a target in *M. leprae*, *M. tuberculosis*, and *Wolbachia endosymbiont* of *Brugia malayi* in the TDR Targets Database [66]. Our analysis also showed that both of these proteins from *Vibrio* are exomembrane/secreted, antigenic, and highly virulent and are therefore suitable for vaccine and drug design where *yajC* is a novel candidate target for *Vibrio* **(Table S2)**.

Apart from these two vaccine targets, the third important target we identified is the membrane bound enzyme *uppP*/*VC0395_A0054* (Undecaprenyl pyrophosphate phosphatase) because of its vital role in the bacterial-specific peptidoglycan biosynthesis pathway and its involvement in cell wall biosynthesis. *uppP* is reported as an antibiotic resistant gene [67] and is also a listed target for *M. Leprae* and *M*. *tuberculosis* in the TDR Targets Database [66]. However, we are reporting *uppP* for the first time as a target in *Vibrio,* therefore it is a novel target for this pathogen.

We used 21 *Vibro* species (both pathogenic and non-pathogenic) available in NCBI and when we searched these three targets (*ompU*, *yajC*, and *uppP*) for their presence among these *Vibrio* species using comparative BLASTp in NCBI server, we found that all three targets are present in 12 species, including the virulent strains *Vibrio anguillarum 775, Vibrio cholerae O1 biovar El Tor str. N16961, Vibrio splendidus LGP32, Vibrio cholerae O395,* and *Vibrio harveyi*, and the non-virulent strain *Vibrio fischeri ES114.* Therefore, all of these selected targets can be used for broad-spectrum drug and vaccine design for a number of *Vibrio* serotypes **(Table S2)**.

### Additional Evaluation of the Essentiality Parameters of Targets

The identified targets *ompU*, *yajC*, and *uppP* were further verified with additional parameters for essentiality in the pathogen genome. Essential enzymes are better targets [15], and most of the essential enzymes belong to the following enzyme classes: transferases, oxidoreductases, ligases, hydrolases, lyases, and isomerases [68]. Among the three targets, *uppP* (EC = 3.6.1.27) is a hydrolase and therefore meets the criteria to be an essential gene. The other two proteins (the secreted protein *ompU* and the preprotein translocase subunit *yajC*) are not enzymes; thus, additional analyses for essentiality were done using a combination of strand-bias, CAI, and COG-bias analysis. The strand-bias analysis showed that these three targets are located in the leading strand and that the codon adaptation indexes (CAI) are 0.63, 0.58, and 0.80, respectively, for *ompU*, *yajC*, and *uppP*, satisfying the cutoff value of >0.5 for being an essential gene. Previous reports have suggested that the essential genes of *M. ulcerans* belong to COG subcategories E, H, J. D, N, V and M [68]. Our identified targets *ompU*, *yajC*, and *uppP*, respectively, belong to M (cell envelope and membrane biogenesis), N (cell motility and secretion), and V (cellular processes and signaling) categories. Therefore, these three targets are essential as per the COG-bias analysis also.

### Evaluation of the Prioritization Parameters of Targets

Proteins with molecular weight ≤110 kDa are proposed to be effective targets [68]. The molecular weights of *Yajc*, *uppP*, and *ompU* are 11.9 kDa, 29,3 kDa, and 37,7 kDa, respectively; therefore, these proteins are of the low molecular weight. This parameter is highly desirable for a target so that the target can be easily purified for further validation [69]. Targets are preferably druggable [70], and 3D structure is required for *in silico* drug discovery by modeling, virtual screening, and druggability analysis. The druggability of these three targets was first tested using a DrugBank search to determine if specific compounds are available against these targets. The results showed that only *ompU* is potentially druggable by small molecules such as N-(6,7,9,10,17,18,20,21-octahydrodibenzo[b,k] [1], [4], [7], [10], [13], [16]hexaoxacyclooctadecin-2-yl) acetamide, Dodecane, and (Hydroxyethyloxy)Tri(Ethyloxy)Octane, N-Octyl-2-Hydroxyethyl Sulfoxide. However, the *E-values* were high. No molecule was found to target *Yajc* and *uppP* in DrugBank. The druggability analysis using DrugBank was negative, potentially because of the novel nature of these identified targets, the non-availability of their 3D structures in PDB, and no previous study on their druggability aspects. Therefore, in this study, we attempted to model these three targets and further tested for druggability using virtual screening.

### PPIs and Host Pathogen Interactions

We used all 10 initially identified targets, including the hypothetical proteins, to make PPI networks of the targets in *V. cholera O395*. The phylogenetic analysis and domain fusion-based PPI networks show that with the exception of *VC0395_0360* (putative hydrolase/Hypothetical) and *LysE*, all targets interact with other *Vibrio* proteins. The KEGG-based analysis of the PPI networks reveals that the selected targets *ompU*, *yajC*, and *uppP* are involved in the *V. cholera* pathogenic cycle, bacterial secretion system, and peptidoglycan biosynthesis pathways, respectively. All of these pathways are unique to bacteria and involved in pathogenesis. Therefore, the PPIs-based analysis also supports our selected final three targets.

**Figure 1:**
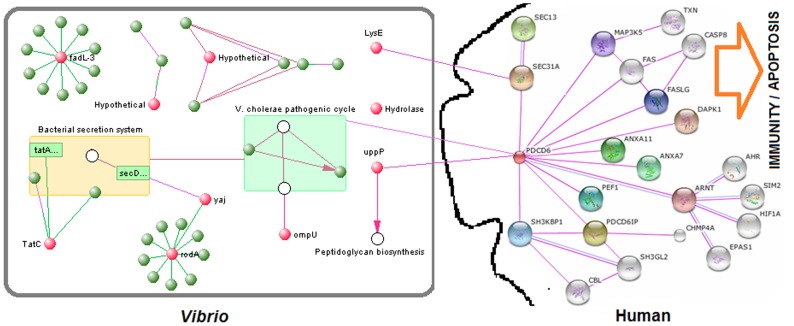
Protein-protein and host-pathogen interactions among ten preliminary identified *Vibrio* targets. The interactions demonstrate that the finally selected three targets (*ompU*, *yajC*, and *uppP*) are involved in *Vibrio* pathogenesis and modulate host response (immunity and apoptosis) by interacting with the host protein PDCD6.

Our host-pathogen interaction analysis revealed that only *uppP* and *LysE* have host protein interacting counterparts. Our selected target *uppP*, which is involved in the peptidoglycan biosynthesis pathway, directly interacts with or binds to the PDCD6 (Programmed cell death 6) protein of the human host. The gene enrichment, pathway, and centrality analyses show that PDCD6 is a key molecule in host immunity and the apoptosis pathway **(** **Figure 1** **)**. The other target, *LysE*, interacts with the host SEC31A (Protein transport protein SEC31A). SEC31A is also involved in immunity and apoptosis in the host but is not a key molecule in these pathways. *LysE* is also not involved in any bacterial pathogenesis pathway, and the exclusion of *LysE* from the final list of targets is therefore justified. Although the two selected targets *ompU* and *yajC* do not directly interact with any host protein, the network analysis showed that the pathways in which these two proteins are involved (the bacterial secretion system and the *V. cholerae* pathogenic cycle) are interlinked and that some proteins in the *V. cholerae* pathogenic cycle interact with PDCD6 **(** **Figure 1** **)**. Therefore, these two selected targets (*ompU* and *yajC*) indirectly interact with PDCD6, leading us to the observation that all of our final selected targets (*ompU*, *yajC*, and *uppP*) interact with PDCD6 and modulate host response in terms of modulation of immunity and the apoptosis pathway in the host.

### 3D Modeling

**Figure 2:**
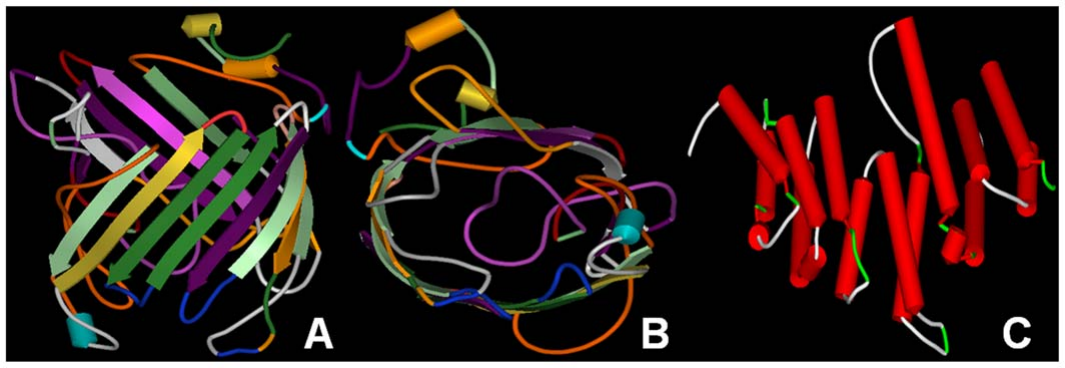
3D models of *Vibrio* targets constructed using threading approaches. A) Front view of *ompU*, B) Side view of *ompU*, and C) Front view of *uppP*.

We first attempted to model *ompU*, *yajC*, and *uppP* using the Phyre 2 server. However, the attempt failed because of unavailability of the proper template. We therefore developed threading-based 3D structure models of these proteins. We were able to model *ompU* and *uppP* using I-TASSER; however, we could not model the *yajC* protein using this approach. Models were validated using the RAMPAGE, ERRAT plot, and Dali servers. Models were found to satisfy all criteria **(Table S3, A-E)**. The 3D models of *uppP* and *ompU* are represented in **Figure 2**.

### Epitope Design

#### Antigenicity and cell-exposed sequences

A good epitope should be cell exposed and antigenic. Therefore, these three targets *ompU*, *yajC*, and *uppP* were first analyzed using VaxiJen and then by TMHMM. The antigenicity scores of these three proteins were found to be 0.766, 0.744 and 0.484, respectively, for *ompU*, *yajC*, and *uppP*; therefore, they are all highly antigenic **(Table S2, column-4)**. The TMHMM-based exomembrane region for *ompU* is 1–350 amino acids and is therefore fully exposed to the outside of the membrane. The cell-exposed amino acid sequences of *uppP* are 30–84, 132–156 and 206–219, and for *yajC*, the sequence is 1–14 **(Table S4, column 6)**.

#### Antigenic B-cell epitope-derived T-cell epitopes

**Figure 3:**
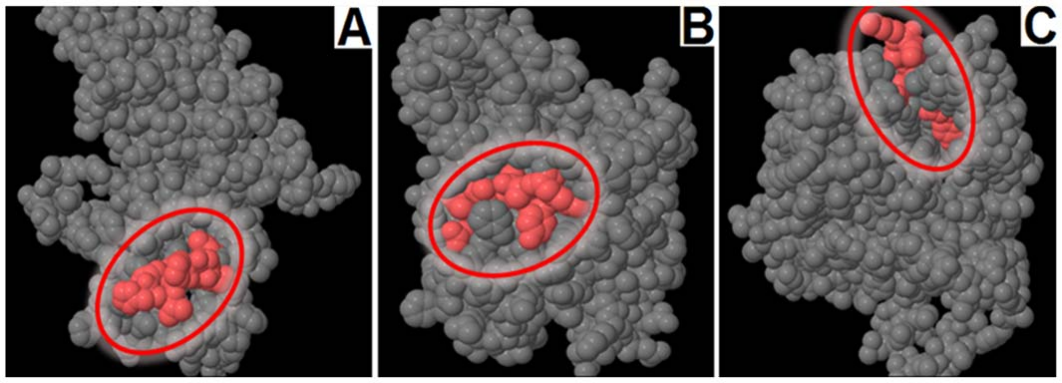
Pepitope analysis of identified T-cell epitopes for their exomembrane topology (colored in red) within the corresponding folded proteins. A) The “VTSGEPVHS” epitope of *uppP*, B) the “VTETNAAKY” epitope of *ompU*, and C) the “YNNAETAKK” epitope of *ompU*.

Using the approach described above, we identified one B-cell epitope from *yajC*, two from *uppP*, and thirteen from *ompU* **(Table S4, column 2)**. However, when we analyzed for the presence of T-cell epitopes within these B-cell epitopes according to our selected criteria, *yajC* did not produce any T-cell epitope. *ompU* generated two (“VTETNAAKY” and “YNNAETAKK”) and *uppP* only one (“VTSGEPVHS”) epitope satisfying all of our criteria **(Table S5)**. The entire protein sequence of *uppP* is non-virulent, but this single epitope is highly virulent and antigenic. Therefore, the *uppP* protein is a candidate novel vaccine target for *Vibrio.* The Pepitope analysis also showed that all of the identified T-cell epitopes are of the exomembrane topology within their corresponding folded proteins **(** **Figure 3** **)**.

### Drug Target and Virtual Screening

**Figure 4:**
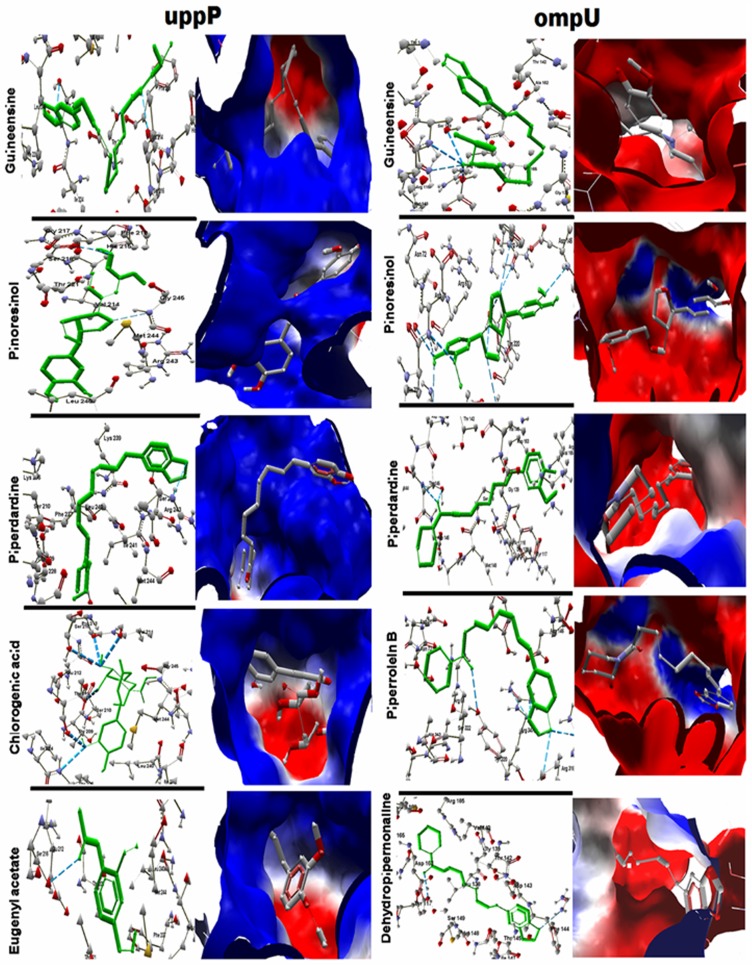
The best seven *Piper betel* compounds that may render activities of *Vibrio* targets *ompU* and *uppP*. GOLD fitness and Moldock scores were considered to select the compounds. Guineesine, Pinoresinol, and Piperdardine inhibit both targets. Dehydropipernonaline and Piperrolein B are effective on *ompU*. Chlorogenic acid and Eugenyl acetate are good ligands for *uppP*.

Since *ompU* is a secreted and *uppP* is an exomembrane protein, they are also suitable drug targets. The *Piper betel* leaf is used in folk medicine for treatment of several situations [72], and the leaf extracts are experimentally shown to be useful as antimicrobial [73], anti-leishmanial [74], antimalarial [75], anti-filarial [76], anti-fungal [77], anti-allergic [78], immunomodulator [79], gastroprotective [80], antioxidant [81], and anti-inflammatory [82] agents. We performed literature mining and collected 128 active phytochemicals from *betel* leaf and used them to screen against these two targets. The docking was done against the best cavity according to the Molegro virtual docker (MVD), CASTp, Pocketfinder, and Active Site Prediction Server **(Table S6)**. The docking results based on the GOLD fitness score and Moldock score show that Guineesine, Pinoresinol, and Piperdardine can bind and render the activities of both the targets with high specificity. Apart from these three common compounds, Dehydropipernonaline and Piperrolein B were found to be effective on *ompU* and Chlorogenic acid and Eugenyl acetate on *uppP* **(** **Figure 4** **, Table S7A)**. Several other *betel* compounds such as Piperardine and Peridine are also found to be effective against these targets however their GOLD fitness and Moldock scores are less. It should also be noted from the docking results that the *Piper betel* compounds are superior to the conventional antibiotics that are prescribed for the treatment of cholera in inhibiting these two targets **(Table S7B)**.

### Validation of Epitopes and Betel Compounds

Among the identified three candidate peptide vaccines, we found *ompU* derived “VTETNAAKY” is 80% identical to an experimentally validated linear peptide vaccine derived from adhesin P1 of *Mycoplasma pneumoniae M129* [71]. However, we could not get any similar peptide in IEDB for other two identified epitopes ((*ompU* derived “YNNAETAKK” and *uppP* based “VTSGEPVHS”), perhaps due to unavailability of similar peptides in IEDB or because of their novelty as candidate vaccines.

**Figure 5:**
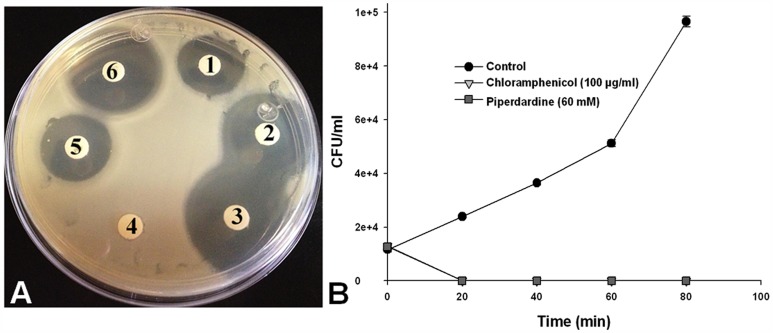
Anti-*Vibrio* activity of Piperdardine. A). Growth inhibition effects Piperdardine, Ampicillin, and Chloranphenicol on *V. Cholerae O1 Inaba* growth as per the disk diffusion method. 1) 100 mM, 2) 200 mM, and 3) 300 mM Piperdardine; 4) water; 5) Ampicillin (10 µg); and 6) Chloranphenicol (30 µg). The zones of inhibition (mm) around disks containing Piperdardine are concentration-dependent: 1) 19.3±0.03; 2) 26.23±0.1; 3) 28.65±0.16. Controls: 4) 0±0; 5) 18.51±0.16; and 6) 29.47±0.16. B). Effects on Piperdardine and Chloranphenicol on *V. Cholerae O1 Inaba* growth as per the Colony-forming units (CFU/ml) assay. As per the method described in the text, 60 mM of Piperdardine (squares) shows anti-*Vibrio* effect similar to 100 µg/ml of Chloramphenicol (triangles).

Piperdardine was used in this preliminary validation. This *betel* compound is found to be highly effective against *V. cholerae O1 Inaba* and the effect is concentration-dependent **(** **Figure 5A** **)**. While we tested Piperdardine for its efficacy in respect to Chloramphenicol using growth kinetics assay, we observed that 60 mM of Piperdardine was able to inhibit *V. cholerae O1 Inaba* growth similar to100 µg/ml of Chloramphenicol treatment **(** **Figure 5B** **)**. Form these assays; it’s also evident that the anti-*Vibrio* efficacy of Piperdardine is better than that of Chloramphenicol, although Piperdardine requires a higher concentration. In this study, we did not check the target specificity of Piperdardine in *V. cholerae O1 Inaba*. However, currently we are conducting in-depth validations and target specificities of all identified *betel* compounds against a number of *Vibrio* species.

### Conclusion

In summary, in this analysis, we have identified *ompU*, *uppP*, and *yajC* from the *Vibrio cholerae* strain *O395* secretome and membrane proteome as novel targets that can be useful in designing broad-spectrum peptide vaccines or drugs against most of the virulent strains of the pathogen. YNNAETAKK and VTETNAAKY from *ompU* and VTSGEPVHS from *uppP* were found to be effective candidate peptide vaccines. *Piper betel*-derived Piperdardine, Pinoresinaol, and Guineensine can target both *ompU* and *uppP*, whereas Dehydropipernonaline and Piperrolein B are specific inhibitors of *ompU* and Eugenyl acetate and Chlorogenic acid are specific to *uppP*. Most of these compounds show better efficacy than the currently-used anti-*Vibrio* drugs in our *in silico* analysis. Our validation results first time demonstrate that Piperdardine exhibits anti-*Vibrio* effects in a dose dependent manner and 60 mM of Piperdardine is having similar anti-Vibrio effect as 100 µg/ml of Chloramphenicol has. We are currently validating all of our identified targets, candidate peptide vaccines, and *betel* derived lead compounds against most of the *Vibrio* strains and serotypes available.

## Supporting Information

Table S1 **Final statistics of membrane and secreted essential proteins.** The proteome of the *Vibrio cholerae* strain *O395* was screened using CELLO, PSLpred, PSORTb, SOSUI-GramN, and SurfG^+^ to identify the membrane proteome and secretome. The genome contains a total of 3998 genes encoding 3875 proteins. The essentialities of these membrane and secreted proteins were determined by DEG-based BLASTp. The cutoff values for bit score, *E-value*, and percentage of identity at the amino acid level, respectively, were ≥100, *E = 0.0001*, and ≥40%. A total of 178 essential proteins were identified in which 119 are membrane located and 59 are secreted. Essential non-host homologs of the pathogen were identified using NCBI Human BLASTp with default parameters. A total of 10 (7 membrane and 3 secreted) essential non-host homologs was found.
(DOC)

Table S2 **Features of the identified 10 targets in** ***V. Cholerae*** **.** Ten *V. cholerae O395* targets were selected based on subtraction proteomics. *VC0395_0360* and *VC0395_0374* are located in Chromosome-I (Ch-I), whereas the other eight targets are located in Chromosome-II (Ch-II). Column-1 and Column-3, respectively, represent locus tags and target names. The blue-colored (*ompU*, *uppP* and *yajC*) meet all conditions for good targets and may be used for broad-spectrum drug and vaccine designing. These three targets are also common to twelve *Vibrio* species. Column 4 represents the COG categories. Column 5 provides detailed annotation of the corresponding *Vibrio* target. Column 6 provides the information on Virulence based on VirulentPred. VaxiJen-based antigenicity of the target *Vibrio* protein is provided in Column 7. Column 8 provides PARTIC and other analysis-based host proteins that interact with the corresponding targets. Columns 9–29 represent *Vibrio* strains/species tested for having identical targets in their genome/proteome based on homology. X represents absence of the target and √ represents presence. The last column represents the BLAST results of corresponding *Vibrio* targets with the human genome/proteome, and all targets show non-homology.
(DOC)

Table S3 A) Template and structure selection for modeling. Because the homology-based approaches for 3D modeling failed, we performed modeling using a threading approach. The three target proteins were submitted to the I-TASSER server, and we observed that the C-score (−5, 2) and TM score were in acceptable ranges. (i) Template selection for modeling Column-1 (The rank of templates) represents the top ten threading templates used by I-TASSER. Ident1 (Column-3) is the percentage sequence identity of the templates in the threading-aligned region with the query sequence. The Ident2 (Column-4) is the percentage sequence identity of the entire template with the query sequence. Coverage (Column-5) represents the coverage of the threading alignment and is equal to the number of aligned residues divided by the length of the query protein. Column-6 represents the normalized *Z-score* of the threading alignments. Alignment with a normalized *Z-score* >1 indicates a good alignment. (ii) Target protein structure selection B) Energy of the protein-modeled structures The modeled structures were subjected to energy minimization. We performed energy minimization in the Swiss PDB Viewer and then checked using RAMPAGE and ERRAT plot. The energies of these two proteins were as follows. C) RAMPAGE results To validate the stereochemical properties of the two targets’ modeled proteins, we used the RAMPAGE server. The expected percentages for residues in the favored region, allowed region, and outliers region are 98%, 2% and 0%, respectively. Our results demonstrated that the parameters of our modeled proteins are close to these cutoff values, and the models are therefore acceptable. D) ERRAT plot results for *ompU* and *uppP*. To further examine the non-bonded interaction of atoms in the models of the two targets, we used the Erraplot server. This server provides the quality factor of the modeled structure. Good, high-resolution structures generally produce quality factor values of approximately 95% or higher. For lower resolutions (2.5 to 3A), the average overall quality factor is approximately 91%. The following ERRAT plot criteria clearly show that our modeled proteins are of high quality. E) Validation of structures using the Dali server To provide strong support of the modeled structure, we performed structure-structure alignment in the Dali server and examined the function. We observed *Z-scores* of 2 that were greater than the threshold for a good alignment for both of the modeled proteins. Therefore, the models are acceptable for further structure-based *in silico* analysis.
(DOC)

Table S4 **Identification of B-cell epitopes.** As described in the methods, the amino acid sequences of *yajC*, *uppP* and *ompU* were subjected to the BCPreds server for B-cell epitope identification. The BCPreds and VaxiJen scores and the transmembrane topology for the selected B-cell epitopes from each target are listed in this table.
(DOC)

Table S5 **Selected B-cell epitope-derived T-cell epitopes and their properties**. The method is adopted as described by Barh et al., 2010 [16]. The final selected epitopes are highlighted in red.
(DOC)

Table S6 **Active residues of** ***ompU*** **and** ***uppP*** **in the best cavity.** We predicted the active residues for the largest cavity from Molegro Virtual Docker (MVD), and we verified our predictions with Cast-P, Pocketfinder and Active site prediction server. All predictions were in good agreement with the predicted result of MVD. However, in *uppP*, we observe a Histidine residue that is well known for ligand specification.
(DOC)

Table S7 **Virtual screening for** ***uppP*** **and** ***ompU*** **.** The docking was performed as described in the methods. The top five ligands were selected based on their GOLD fitness score, MolDock score and RMSD. A ligand with a GOLD fitness score >25 is considered to be a good ligand. Similarly, the standard RMSD ranges from 0 to 4. Apart from electrostatic and hydrophobic interactions, more than 2 H-bonds indicate the ligand stability in the docked position.
(DOC)
