# Supplementary material for: Exoproteome and Secretome Derived Broad Spectrum Novel Drug and Vaccine Candidates in Vibrio cholerae Targeted by Piper betel Derived Compounds
Source: PLoS One. 2013 Jan 30;8(1):e52773. doi: 10.1371/journal.pone.0052773 (PMC3559646; doi:10.1371/journal.pone.0052773)
Supplement: Table S1 — Final statistics of membrane and secreted essential proteins. The proteome of the Vibrio cholerae strain O395 was screened using CELLO, PSLpred, PSORTb, SOSUI-GramN, and SurfG+ to identify the membrane proteome and secretome. The genome contains a total of 3998 genes encoding 3875 proteins. The essentialities of these membrane and secreted proteins were determined by DEG-based BLASTp. The cutoff values for bit score, E-value, and percentage of identity at the amino acid level, respectively, were ≥100, E = 0.0001, and ≥40%. A total of 178 essential proteins were identified in which 119 are membrane located and 59 are secreted. Essential non-host homologs of the pathogen were identified using NCBI Human BLASTp with default parameters. A total of 10 (7 membrane and 3 secreted) essential non-host homologs was found. (DOC) [file pone.0052773.s001.doc]

**Table S1**

**Final statistics of membrane and secreted essential proteins.** The proteome of the *Vibrio cholerae* strain *O395* was screened using CELLO, PSLpred, PSORTb, SOSUI-GramN, and SurfG^+^ to identify the membrane proteome and secretome. The genome contains a total of 3998 genes encoding 3875 proteins. The essentialities of these membrane and secreted proteins were determined by DEG-based BLASTp. The cutoff values for bit score, *E-value*, and percentage of identity at the amino acid level, respectively, were ≥100, *E = 0.0001*, and ≥40%. A total of 178 essential proteins were identified in which 119 are membrane located and 59 are secreted. Essential non-host homologs of the pathogen were identified using NCBI Human BLASTp with default parameters. A total of 10 (7 membrane and 3 secreted) essential non-host homologs was found.

| **Chro. No** | **Total No of** | | | | | | | | | |
| --- | --- | --- | --- | --- | --- | --- | --- | --- | --- | --- |
|  | **Genes** | **Protein coding genes** | **Membrane proteins** | **Secreted proteins** | **Essential proteins** | **Secreted essential** | **Membrane essential** | **Secreted essential non-host homolog** | **Membrane**  **essential non-host homolog** | **Essential non-host homolog** |
| Ch-I | 1137 | [1133](http://www.ncbi.nlm.nih.gov/genome?Db=genome&Cmd=Retrieve&dopt=Protein+Table&list_uids=20971) | 160 | 113 | 50 | 15 | 35 | 2 | 0 | 2 |
| Ch-II | 2861 | 2742 | 353 | 204 | 128 | 44 | 84 | 1 | 7 | 8 |
| **Total** | **3998** | **3875** | **513** | **317** | **178** | **59** | **119** | **3** | **7** | **10** |
