# Supplementary material for: Exoproteome and Secretome Derived Broad Spectrum Novel Drug and Vaccine Candidates in Vibrio cholerae Targeted by Piper betel Derived Compounds
Source: PLoS One. 2013 Jan 30;8(1):e52773. doi: 10.1371/journal.pone.0052773 (PMC3559646; doi:10.1371/journal.pone.0052773)
Supplement: Table S2 — Features of the identified 10 targets in V. Cholerae . Ten V. cholerae O395 targets were selected based on subtraction proteomics. VC0395_0360 and VC0395_0374 are located in Chromosome-I (Ch-I), whereas the other eight targets are located in Chromosome-II (Ch-II). Column-1 and Column-3, respectively, represent locus tags and target names. The blue-colored (ompU, uppP and yajC) meet all conditions for good targets and may be used for broad-spectrum drug and vaccine designing. These three targets are also common to twelve Vibrio species. Column 4 represents the COG categories. Column 5 provides detailed annotation of the corresponding Vibrio target. Column 6 provides the information on Virulence based on VirulentPred. VaxiJen-based antigenicity of the target Vibrio protein is provided in Column 7. Column 8 provides PARTIC and other analysis-based host proteins that interact with the corresponding targets. Columns 9–29 represent Vibrio strains/species tested for having identical targets in their genome/proteome based on homology. X represents absence of the target and √ represents presence. The last column represents the BLAST results of corresponding Vibrio targets with the human genome/proteome, and all targets show non-homology. (DOC) [file pone.0052773.s002.doc]

**Table S2**

**Features of the identified 10 targets in *V. Cholerae*.** Ten *V. cholerae O395* targets were selected based on subtraction proteomics. *VC0395_0360* and *VC0395_0374* are located in Chromosome-I (Ch-I), whereas the other eight targets are located in Chromosome-II (Ch-II). Column-1 and Column-3, respectively, represent locus tags and target names. The blue-colored (*ompU*, *uppP* and *yajC*) meet all conditions for good targets and may be used for broad-spectrum drug and vaccine designing. These three targets are also common to twelve *Vibrio* species. Column 4 represents the COG categories. Column 5 provides detailed annotation of the corresponding *Vibrio* target. Column 6 provides the information on Virulence based on VirulentPred. VaxiJen-based antigenicity of the target *Vibrio* protein is provided in Column 7. Column 8 provides PARTIC and other analysis-based host proteins that interact with the corresponding targets. Columns 9-29 represent *Vibrio* strains/species tested for having identical targets in their genome/proteome based on homology. X represents absence of the target and √ represents presence. The last column represents the BLAST results of corresponding *Vibrio* targets with the human genome/proteome, and all targets show non-homology.

| **1** | **2** | **3** | **4** | **5** | **6** | **7** | **8** | **9** | **10** | **11** | **12** | **13** | **14** | **15** | **16** | **17** | **18** | **19** | **20** | **21** | **22** | **23** | **24** | **25** | **26** | **27** | **28** | **29** | **30** |
| --- | --- | --- | --- | --- | --- | --- | --- | --- | --- | --- | --- | --- | --- | --- | --- | --- | --- | --- | --- | --- | --- | --- | --- | --- | --- | --- | --- | --- | --- |
|  |  |  |  |  |  |  |  | **21 Vibrio species (Red and yellow highlighted are virulent strains)** | | | | | | | | | | | | | | | | | | | | |  |
| **locus tags of *V. cholerae O395*** | **UniProt ID** | **Gene/ Protein Symbol and Names** | **COG Classifications** | **Functional Annotation from NCBI, UniProt, KEGG, and various other databases** | **VirulentPred (Virulant-Y/N)** | **Overall Antigen Prediction VaxiJen (Cutoff=0.4)** | **Host (Human) interacting proteins** | *V. anguillarum 775* | *V. cholerae Ban5* | *V. cholerae Ind4* | *V. cholerae Ind5* | *V. cholerae LMA3894-4* | *V. cholerae M66-2* | *V. cholerae MJ-1236* | *V. cholerae Mex1* | ***V. cholerae O1 biovar El Tor str. N16961*** | *V. fischeri ES114* | *V. fischeri MJ11* | *V. fluvialis Ind1* | *V. furnissii NCTC 11218* | *V. harveyi ATCC BAA-1116* | *V. parahaemolyticus RIMD 2210633* | *V. sp. Ex25* | *V. splendidus LGP32* | *V. vulnificus CMCP6* | *V. vulnificus MO6-24/O* | *V. vulnificus YJ016* | ***v. cholerae O395*** | **Human (Host)** |
| VC0395_0360 | A5F0P3 | Putative hydrolase / Hypothetical | M | MF: hydrolase activity BP: Lipid Metabolism CC: secreted | Y | 0.4575 (Antigenic) | NA | X | X | X | X | X | X | X | X | √ | X | √ | X | X | √ | X | X | √ | X | √ | X | √ | X |
| VC0395_0374 | A5F0M2 | **fadL-3**  (long-chain fatty acid transport protein) | I | CC: Cell outer membrane | Y | 0.6131 (Antigenic) | NA | √ | X | X | X | X | X | √ | X | √ | X | X | X | X | √ | √ | √ | √ | X | X | X | √ | X |
| VC0395_A0033 | A5F9F0 | **LysE** / YggA family protein | R | BP: amino acid transport CC: membrane | N | 0.559 (Antigenic) | SC31A  (*B. anthasis* **LysE** interacting) | √ | X | X | X | X | X | X | X | √ | X | X | X | X | √ | √ | √ | √ | X | √ | √ | √ | X |
| VC0395_A0054 | A5F9D4 | **uppP**  (undecaprenyl pyrophosphate phosphatase) | V | MF: undecaprenyl-diphosphatase activity BP: Peptidoglycan biosynthesis, cell wall organization, regulation of cell shape, response to antibiotic CC: plasma membrane Pathway: Peptidoglycan biosynthesis EC:3.6.1.27 | N | 0.4837 (Antigenic) | PDCD6  (*Y. pestis* **ppP**  interacting) | √ | X | X | X | X | X | X | X | √ | √ | √ | X | X | √ | √ | √ | √ | √ | √ | √ | √ | X |
| VC0395_A0162 | A5F934 | **ompU**  (outer membrane protein OmpU) | M | MF: porin activity BP: ion transport CC: Cell outer membrane/ secreted  Pathway: Vibrio cholerae pathogenic cycle | Y | 0.7660 (Antigenic) | NA | √ | X | X | X | X | X | X | X | √ | √ | √ | X | X | √ | √ | √ | √ | √ | √ | √ | √ | X |
| VC0395_A0271 | A5F3H3 | **yajC**  (preprotein translocase subunit YajC) | N | BP: transport CC: Cell outer membrane Pathway: Membrane Transport; Bacterial secretion system | Y | 0.744 (Antigenic) | NA | √ | X | X | X | X | X | X | X | √ | √ | √ | X | X | √ | √ | √ | √ | √ | √ | √ | √ | X |
| VC0395_A0472 | A5F2Y5 | **rodA**  (rod shape-determining protein RodA) | D | BP: cell cycle, regulation of cell shape CC: Cell membrane | N | 0.6224 (Antigenic) | NA | √ | X | X | X | X | X | X | X | √ | √ | √ | X | X | √ | √ | √ | √ | √ | √ | √ | √ | X |
| VC0395_A1375 | A5F7B6 | Hypothetical | S | CC: Cell membrane | Y | 0.4579 (Antigenic) | NA | √ | X | X | X | X | X | X | X | √ | X | √ | X | X | √ | √ | √ | X | X | X | X | √ | X |
| VC0395_A2427 | A5F4G0 | sec-independent protein translocase protein **TatC** / Hypothetical | N | MF: protein transmembrane transporter activity BP: protein transport by the Tat complex CC: Cell membrane Pathway: Membrane Transport; Bacterial secretion system | Y | 0.5546 (Antigenic) | NA | √ | X | X | X | X | X | X | X | √ | √ | √ | X | X | X | √ | √ | √ | √ | √ | √ | √ | X |
| VC0395_A2856 | A5F8P3 | Hypothetical | S | CC: Cell membrane/secreted | Y | 0.3112  (Non-antigenic) | NA | √ | X | X | X | X | X | X | X | √ | √ | √ | X | X | √ | √ | √ | X | √ | √ | √ | √ | X |

**COG Classes- D:** Cell division and chromosome partitioning; **I:** Lipid metabolism; **M:** Cell envelope biogenesis, outer membrane; **N:** Cell motility and secretion; **R:** General function prediction only; **S:** Function unknown; and **V:** Cellular processes and signalling.

NA: Not available. MF: Molecular Function; BP: Biological Process; CC: Cellular Component. X: not present in the strain/species; √: present in the strain/species.
