# Supplementary material for: Exoproteome and Secretome Derived Broad Spectrum Novel Drug and Vaccine Candidates in Vibrio cholerae Targeted by Piper betel Derived Compounds
Source: PLoS One. 2013 Jan 30;8(1):e52773. doi: 10.1371/journal.pone.0052773 (PMC3559646; doi:10.1371/journal.pone.0052773)
Supplement: Table S3 — A) Template and structure selection for modeling. Because the homology-based approaches for 3D modeling failed, we performed modeling using a threading approach. The three target proteins were submitted to the I-TASSER server, and we observed that the C-score (−5, 2) and TM score were in acceptable ranges. (i) Template selection for modeling Column-1 (The rank of templates) represents the top ten threading templates used by I-TASSER. Ident1 (Column-3) is the percentage sequence identity of the templates in the threading-aligned region with the query sequence. The Ident2 (Column-4) is the percentage sequence identity of the entire template with the query sequence. Coverage (Column-5) represents the coverage of the threading alignment and is equal to the number of aligned residues divided by the length of the query protein. Column-6 represents the normalized Z-score of the threading alignments. Alignment with a normalized Z-score >1 indicates a good alignment. (ii) Target protein structure selection B) Energy of the protein-modeled structures The modeled structures were subjected to energy minimization. We performed energy minimization in the Swiss PDB Viewer and then checked using RAMPAGE and ERRAT plot. The energies of these two proteins were as follows. C) RAMPAGE results To validate the stereochemical properties of the two targets’ modeled proteins, we used the RAMPAGE server. The expected percentages for residues in the favored region, allowed region, and outliers region are 98%, 2% and 0%, respectively. Our results demonstrated that the parameters of our modeled proteins are close to these cutoff values, and the models are therefore acceptable. D) ERRAT plot results for ompU and uppP. To further examine the non-bonded interaction of atoms in the models of the two targets, we used the Erraplot server. This server provides the quality factor of the modeled structure. Good, high-resolution structures generally produce quality factor values of approximately 95% or h [file pone.0052773.s003.doc]

**Table S3**

**A) Template and structure selection for modeling**

Because the homology-based approaches for 3D modeling failed, we performed modeling using a threading approach. The three target proteins were submitted to the I-Tasser server, and we observed that the C-score (-5, 2) and TM score were in acceptable ranges.

**(i) Template selection for modeling**

Column-1 (The rank of templates) represents the top ten threading templates used by I-TASSER. Ident1 (Column-3) is the percentage sequence identity of the templates in the threading-aligned region with the query sequence. The Ident2 (Column-4) is the percentage sequence identity of the entire template with the query sequence. Coverage (Column-5) represents the coverage of the threading alignment and is equal to the number of aligned residues divided by the length of the query protein. Column-6 represents the normalized *Z-score* of the threading alignments. Alignment with a normalized *Z-score* >1 indicates a good alignment.

| **Rank** | **PDB Hit** | **Iden1** | **Iden2** | **Coverage** | **Norm. Z-score** |
| --- | --- | --- | --- | --- | --- |
| 1 | [1phoA](http://www.rcsb.org/pdb/explore/explore.do?structureId=1pho) | 0.24 | 0.25 | 0.87 | 2.30 |
| 2 | [1phoA](http://www.rcsb.org/pdb/explore/explore.do?structureId=1pho) | 0.22 | 0.25 | 0.87 | 4.96 |
| 3 | [1hxxA](http://www.rcsb.org/pdb/explore/explore.do?structureId=1hxx) | 0.22 | 0.24 | 0.88 | 7.20 |
| 4 | [3nsgA](http://www.rcsb.org/pdb/explore/explore.do?structureId=3nsg) | 0.23 | 0.26 | 0.87 | 3.22 |
| 5 | [1phoA](http://www.rcsb.org/pdb/explore/explore.do?structureId=1pho) | 0.23 | 0.25 | 0.88 | 2.70 |
| 6 | [2zfgA](http://www.rcsb.org/pdb/explore/explore.do?structureId=2zfg) | 0.23 | 0.26 | 0.88 | 5.10 |
| 7 | [3nsgA](http://www.rcsb.org/pdb/explore/explore.do?structureId=3nsg) | 0.24 | 0.26 | 0.87 | 9.95 |
| 8 | [1pho](http://www.rcsb.org/pdb/explore/explore.do?structureId=1pho) | 0.25 | 0.25 | 0.83 | 3.86 |
| 9 | [1hxxA](http://www.rcsb.org/pdb/explore/explore.do?structureId=1hxx) | 0.22 | 0.24 | 0.87 | 6.24 |
| 10 | [1phoA](http://www.rcsb.org/pdb/explore/explore.do?structureId=1pho) | 0.26 | 0.26 | 0.84 | 5.67 |

**(ii) Target protein structure selection**

| **Proteins** | **C scores** | **TM score** | **Cluster density** |
| --- | --- | --- | --- |
| ***uppP*** | -3.04 | 0.588 | 0.064 |
| ***ompU*** | -1.127 | 0.8467 | 0.0681 |

**B) Energy of the protein-modeled structures**

The modeled structures were subjected to energy minimization. We performed energy minimization in the Swiss PDB Viewer and then checked using Rampage and Errat plot. The energies of these two proteins were as follows.

| **Target proteins** | **Energy values** |
| --- | --- |
| ***ompU*** | -7521.443 KJ/Mol. |
| ***uppP*** | -6315.77 KJ/Mol. |

**C) Rampage results**

To validate the stereochemical properties of the two targets’ modeled proteins, we used the Rampage server. The expected percentages for residues in the favored region, allowed region, and outliers region are 98%, 2% and 0%, respectively. Our results demonstrated that the parameters of our modeled proteins are close to these cutoff values, and the models are therefore acceptable.

| **Parameters** | **ompU** | **uppP** |
| --- | --- | --- |
| Number of residues in favored region (expected is 98%) | 97.6% | 96.9% |
| Number of residues in allowed region (expected is 2%) | 3.4% | 3.2% |
| Number of residues in outlier region (expected is 0%) | 0.4% | 0.6% |

**D) Errat plot results for *ompU* and *uppP***

To further examine the non-bonded interaction of atoms in the models of the two targets, we used the Erraplot server. This server provides the quality factor of the modeled structure. Good, high-resolution structures generally produce quality factor values of approximately 95% or higher. For lower resolutions (2.5 to 3A), the average overall quality factor is approximately 91%. The following Errat plot criteria clearly show that our modeled proteins are of high quality.

| **Target proteins** | **Quality factor values** |
| --- | --- |
| ***ompU*** | 90.76 |
| ***uppP*** | 99.23 |

**E) Validation of structures using the Dali server**

To provide strong support of the modeled structure, we performed structure-structure alignment in the Dali server and examined the function. We observed *Z-scores* of 2 that were greater than the threshold for a good alignment for both of the modeled proteins. Therefore, the models are acceptable for further structure-based *in silico* analysis.

| **Chain** | **Z-Score** | **RMSD** | **Activity** |
| --- | --- | --- | --- |
| ***uppP*** (undecaprenyl pyrophosphate phosphatase) | | | |
| 2iae-E | 12.4 | 4.1 | Serine/Threonine –Protein Phosphatse 2A 65KDA |
| 2npp-D | 11.4 | 3.4 | Protein Phosphatase 2,Regulatory subunit A |
| ***ompU*** (outer membrane protein OmpU) | | | |
| 1mpf-A | 23.9 | 2.8 | Matrix Porin Outer Membrane Protein |
| 2zfg-A | 23.9 | 2.8 | Outer membrane protein F |
