# Supplementary material for: Exoproteome and Secretome Derived Broad Spectrum Novel Drug and Vaccine Candidates in Vibrio cholerae Targeted by Piper betel Derived Compounds
Source: PLoS One. 2013 Jan 30;8(1):e52773. doi: 10.1371/journal.pone.0052773 (PMC3559646; doi:10.1371/journal.pone.0052773)
Supplement: Table S4 — Identification of B-cell epitopes. As described in the methods, the amino acid sequences of yajC, uppP and ompU were subjected to the BCPreds server for B-cell epitope identification. The BCPreds and VaxiJen scores and the transmembrane topology for the selected B-cell epitopes from each target are listed in this table. (DOC) [file pone.0052773.s004.doc]

**Table S4**

**Identification of B-cell epitopes.** As described in the methods, the amino acid sequences of *yajC*, *uppP* and *ompU* were subjected to the BCPreds server for B-cell epitope identification. The BCPreds and VaxiJen scores and the transmembrane topology for the selected B-cell epitopes from each target are listed in this table.

| **Name of proteins and Vaxigen scores** | **BCPreds based B-cell epitope sequences** | **Amino acid positions** | **BCPreds scores** | **Vaxigen score of B-cell epitopes** | ****IEDB**  **at 70% identity**  **(Yes/ No)** | **TMHMM based surface exposed amino acid positions** |
| --- | --- | --- | --- | --- | --- | --- |
| **Yajc**  (protein translocase subunit)  **(0.744)** | AHAAGEGAPQGGGFEMIIML | 7 | 1 | 1.4406 | Y  (B- & T-cell) | 1 to 14 |
| **ompU**  (outer membrane protein)  **(0.7660)** | YTFGDTGFNVGAGYADQDDQ | 221 | 0.995 | 0.5918 | Y (B-cell) | 1-350 |
|  | NVVTETNAAKYSDNGEDGYS | 197 | 0.994 | 1.1706 | Y (B-cell) | 1-350 |
|  | ATGAYADGINQSGDKAGSTV | 25 | 0.993 | 1.4842 | Y (B-cell) | 1-350 |
|  | FTTNDQGKNASNNSLDNRYT | 99 | 0.979 | 1.3996 | Y (B- & T-cell) | 1-350 |
|  | SLKDGKAQDNSRVRLNFLGK | 63 | 0.923 | 1.0601 | Y (T-cell) | 1-350 |
|  | GQFGDLGVKASYRFADRNAV | 173 | 0.814 | 0.6492 | Y  (B- & T-cell) | 1-350 |
|  | TATYNNAETAKKTSADNFAI | 288 | 0.799 | 1.1033 | Y  (B- & T-cell) | 1-350 |
|  | QSGDKAGSTVYSAKGTSLEV | 35 | 1 | 1.658 | Y (B-cell) | 1-350 |
|  | MGNVVTETNAAKYSDNGEDG | 195 | 1 | 1.1666 | Y (B-cell) | 1-350 |
|  | YNNAETAKKTSADNFAIDAT | 291 | 1 | 1.2066 | Y  (B- & T-cell) | 1-350 |
|  | LKDGKAQDNSRVRLNFLGKA | 64 | 1 | 1.2329 | Y (T-cell) | 1-350 |
|  | LSAIYTFGDTGFNVGAGYAD | 217 | 1 | 0.2865 | N | 1-350 |
|  | **FTTNDQGKNASNNSLDNRYT** | 99 | 0.998 | 1.3996 | Y  (B- & T-cell) | 1-350 |
| **uppP**  (undecaprenyl pyrophosphate phosphatase)  **(0.4837)** |  |  |  |  |  |  |
|  | VTSGEPVHSGFLLTGIITSF | 208 | 1 | 0.0013 | Y (B-cell) | 206-219 |
|  | **GDRSKEAKLAWLIVLATIPA** | 77 | 0.999 | 1.0066 | Y  (B- & T-cell) | 30-84 |

Reds are selected for T-epitopes; Bold: having high no of both B- & T- cell epitopes; **Based on experimental validation
