# Supplementary material for: Exoproteome and Secretome Derived Broad Spectrum Novel Drug and Vaccine Candidates in Vibrio cholerae Targeted by Piper betel Derived Compounds
Source: PLoS One. 2013 Jan 30;8(1):e52773. doi: 10.1371/journal.pone.0052773 (PMC3559646; doi:10.1371/journal.pone.0052773)
Supplement: Table S5 — Selected B-cell epitope-derived T-cell epitopes and their properties. The method is adopted as described by Barh et al., 2010 [16]. The final selected epitopes are highlighted in red. (DOC) [file pone.0052773.s005.doc]

**Table S5**

Selected B-cell epitope-derived T-cell epitopes and their properties. The method is adopted as described by Barh et al., 2010 [16]. The final selected epitopes are highlighted in red.

| **B cell epitopes**  **(****IEDB at 70% identity (Yes/ No): Based on assay) | **T epitope based on Propred I and Propred** | ****IEDB**  **at 70% identity**  **(Yes/ No)** | **Total no. MHC binding alleles** | **Vaxijen Scores** | **TMHMM Position of the T-cell epitope** | **MHCPred based IC_50_ values** | | **T-epitope designer scores** | | | **Number of alleles binding in T-cell epitope designer** | **T-epitope designer Percentage (>75%)** | **Cluster scores as per the Pepitope analysis** |
| --- | --- | --- | --- | --- | --- | --- | --- | --- | --- | --- | --- | --- | --- |
|  |  |  |  |  |  | DRB1*0101 | DRB1*401 | A*0201 | A*0204 | B*2705 |  |  |  |

ompU

| YTFGDTGFNVGAGYAD  Y (B-cell) | YTFGDTGFN | N | 12 | 0.3628 | 221-229 | 142.89 | 136.46 | -97.78 | -374.41 | 1028.21 | 742 | 742/809  (91.78%) |  |
| --- | --- | --- | --- | --- | --- | --- | --- | --- | --- | --- | --- | --- | --- |
| **FTTNDQGKNASNNSLDNRYT**  Y (B- & T-cell) | FTTNDQGKN | N | 4 | 2.2081 | 99-107 | 9.4 | 306.9 | 48.33 | -623.01 | 1629.52 | 731 | 731/809  (90.35%) |  |
|  | FTTNDQGKN | N | 4 | 2.2081 | 99-107 | 9.4 | 306.9 | 48.33 | -623.01 | 1629.52 | 731 | 731/809  (90.35%) |  |
| **NVVTETNAAKYSDNGEDG**  Y (B-cell) | **VTETNAAKY** | Y  (B-cell) | **14** | **1.3815** | **199-207** | **26.18** | **580.76** | **278.28** | **148.69** | **700.31** | **804** | **804/809**  **(99.38%)** | **Best cluster Score:11.581, Residue number: 9** |
|  | VVTETNAAK | Y  (B-cell) | 35 | 1.4364 | 198-206 | 278.61 | 276.06 | 418.6 | 931.87 | 61.52 | 282 | 282/809  (34.85%) |  |
| **YNNAETAKKTSADNFAI**  Y (B- & T-cell) | **YNNAETAKK** | **N** | **52** | **1.594** | **291-299** | **15.38** | **377.57** | **632.56** | **48.76** | **1611.67** | **804** | **804/809 (99.38%)** | **Best cluster**  **Score: 12.766**  **Residues number: 9** |

uppP

| **VTSGEPVHSGFLLTGIITSF**  Y (B-cell) | **VTSGEPVHS** | **N** | **13** | **1.238** | **208 - 216** | **571.48** | **763.84** | **-735.8** | **-1278.3** | **-134.3** | **219** | **219/809**  **(27.07%)** | **Best cluster**  **Score: 16.175**  **Residues number: 9** |
| --- | --- | --- | --- | --- | --- | --- | --- | --- | --- | --- | --- | --- | --- |

**Based on experimental validation
