# Supplementary material for: Exoproteome and Secretome Derived Broad Spectrum Novel Drug and Vaccine Candidates in Vibrio cholerae Targeted by Piper betel Derived Compounds
Source: PLoS One. 2013 Jan 30;8(1):e52773. doi: 10.1371/journal.pone.0052773 (PMC3559646; doi:10.1371/journal.pone.0052773)
Supplement: Table S6 — Active residues of ompU and uppP in the best cavity. We predicted the active residues for the largest cavity from Molegro Virtual Docker (MVD), and we verified our predictions with Cast-P, Pocketfinder and Active site prediction server. All predictions were in good agreement with the predicted result of MVD. However, in uppP, we observe a Histidine residue that is well known for ligand specification. (DOC) [file pone.0052773.s006.doc]

**Table S6**

**Active residues of *ompU* and *uppP* in the best cavity.** We predicted the active residues for the largest cavity from Molegro Virtual Docker (MVD), and we verified our predictions with Cast-P, Pocketfinder and Active site prediction server. All predictions were in good agreement with the predicted result of MVD. However, in *uppP*, we observe a Histidine residue that is well known for ligand specification.

| **Targets** | **Cavity volume** | **Active Sites** |
| --- | --- | --- |
| ***ompU*** | 3703.02 | Arg116, Arg185, Asp163, Gly139, Ile147, Leu138, Met148, Ser149, Thr142, Tyr117, Val140 |
| ***uppP*** | 132.096 | Val214, Ile228, Cys233, Val255, Leu254, Ile251, Arg253, Thr225, Ser210, Gly257, His215, Pro248. |
