# Supplementary material for: Exoproteome and Secretome Derived Broad Spectrum Novel Drug and Vaccine Candidates in Vibrio cholerae Targeted by Piper betel Derived Compounds
Source: PLoS One. 2013 Jan 30;8(1):e52773. doi: 10.1371/journal.pone.0052773 (PMC3559646; doi:10.1371/journal.pone.0052773)
Supplement: Table S7 — Virtual screening for uppP and ompU . The docking was performed as described in the methods. The top five ligands were selected based on their GOLD fitness score, MolDock score and RMSD. A ligand with a GOLD fitness score >25 is considered to be a good ligand. Similarly, the standard RMSD ranges from 0 to 4. Apart from electrostatic and hydrophobic interactions, more than 2 H-bonds indicate the ligand stability in the docked position. (DOC) [file pone.0052773.s007.doc]

**Table S7**

**Virtual screening for *uppP* and *ompU*.** The docking was performed as described in the methods. The top five ligands were selected based on their Gold fitness score, MolDock score and RMSD. A ligand with a Gold fitness score > 25 is considered to be a good ligand. Similarly, the standard RMSD ranges from 0 to 4. Apart from electrostatic and hydrophobic interactions, more than 2 H-bonds indicate the ligand stability in the docked position.

**A) Top 5 *Piper betel* compounds**

| **Targets** | **Ligands** | **CCDC GOLD 4.1.2** | | | | | **Molegro Virtual Docker 4.2.0** | | | **Hydrogen**  **Bonds** |
| --- | --- | --- | --- | --- | --- | --- | --- | --- | --- | --- |
|  |  | **S(hb_ext)** | **S(vdw_ext)** | **S(hb_int)** | **S(int)** | **GOLD fitness Scores** | **MolDock Score** | **RMSD** | **Torsion** |  |
| **ompU** | Guineesine | 2 | 49.54 | 0 | -14.01 | 56.11 | -137.14 | 3.64 | 12 | 4 |
|  | Dehydropipernonaline | 6.75 | 42.72 | 0 | -10.98 | 54.49 | -118.62 | 1.71 | 6 | 2 |
|  | Piperrolein B | 7.04 | 40.05 | 0 | -9.67 | 52.44 | -111.9 | 2.75 | 8 | 5 |
|  | Piperdardine | 2.97 | 42.3 | 0 | -9.1 | 52.04 | -133.74 | 0.45 | 6 | 2 |
|  | Pinoresinol | 5.62 | 37.64 | 0 | -8.58 | 48.79 | -137.54 | 0.23 | 4 | 10 |
| **uppP** | Chlorogenic acid | 8.78 | 34.86 | 0 | -15.86 | 40.85 | -138.57 | 1.689 | 5 | 6 |
|  | Guineesine | 0 | 46.75 | 0 | -9.22 | 55.05 | -155.91 | 1.52 | 12 | 3 |
|  | Pinoresinol | 2 | 41.3 | 0 | -9.96 | 48.83 | -136.86 | 0.26 | 4 | 2 |
|  | Piperdardine | 4.27 | 41.23 | 0 | -9.83 | 51.12 | -128.11 | 0.39 | 6 | 2 |
|  | Eugenyl acetate | 1.8 | 34.73 | 0 | -5.3 | 44.26 | -96.25 | 0.1 | 5 | 1 |

**B) Top 5 antibiotics**

| **Targets** | **Antibiotics** | **CCDC GOLD 4.1.2** | | | | | **Molegro Virtual Docker 4.2.0** | | | **Hydrogen**  **Bonds** |
| --- | --- | --- | --- | --- | --- | --- | --- | --- | --- | --- |
|  |  | **S(hb_ext)** | **S(vdw_ext)** | **S(hb_int)** | **S(int)** | **GOLD fitness Scores** | **MolDock Scores** | **RMSD** | **Torsion** |  |
| **uppP** | Ciprofloxacin | 0 | 40.99 | 0 | -5.36 | 51 | -111.19 | 0.8 | 10 | 12 |
|  | Chloramphenicol | 4.77 | 35.6 | 0 | -3.69 | 50.04 | -105.55 | 0.6 | 5 | 9 |
|  | Ampicillin | 1.16 | 38.93 | 0 | -6.19 | 48.5 | -123.93 | 0.9 | 7 | 9 |
|  | Trimethoprim | 2.86 | 40.48 | 0 | -9.76 | 48.76 | -111.24 | 0.01 | 8 | 12 |
|  | Norfloxacin | 1.22 | 40.39 | 0 | -10.48 | 46.27 | -99.79 | 0.6 | 6 | 9 |
| **ompU** | Ampicillin | 7.37 | 34.21 | 0 | -1.15 | 53.27 | -115.52 | 0.5 | 9 | 6 |
|  | Chloramphenicol | 5.01 | 32.08 | 0 | -4.06 | 45.07 | -86.75 | 6.5 | 3 | 6 |
|  | Furazolidone | 13.17 | 24.23 | 0 | -1.7 | 44.79 | -123.34 | 0.02 | 5 | 4 |
|  | Ciprofloxacin | 2 | 34.63 | 0 | -5.38 | 44.24 | -88.16 | 0.9 | 4 | 8 |
|  | Trimethoprim | 6.24 | 32.6 | 0 | -8.66 | 42.4 | -87.93 | 2.56 | 7 | 5 |
